# Cultural adaptation and Sepedi translation of the Activities-specific Balance Confidence scale

**DOI:** 10.4102/sajcd.v71i1.1004

**Published:** 2024-05-21

**Authors:** Tammy L. Prinsloo, Karin Joubert

**Affiliations:** 1 Department of Audiology, Faculty of Humanities, University of the Witwatersrand, Johannesburg, South Africa

**Keywords:** falls, aged, ABC scale, activities and participation, cultural adaptation, linguistic appropriateness

## Abstract

**Background:**

The Activities-specific Balance Confidence (ABC) scale is a widely used measure to identify older adults with balance difficulties. However, its applicability in the diverse South African context is hindered by cross-cultural and linguistic differences. Limited research exists on the use of the ABC scale in native South African languages.

**Objectives:**

This study aimed to translate and culturally adapt the ABC scale into Sepedi, evaluate its reliability and determine self-perceived balance confidence among elderly individuals in a rural community.

**Method:**

The ABC scale was translated and culturally adapted into Sepedi. Two trained raters administered the Sepedi version of the ABC (ABC-S) scale to 32 individuals aged between 60 and 88 years. Test-retest reliability and inter-rater reliability were determined, with one rater re-administering the scale 2 weeks later.

**Results:**

Ten items from the original ABC scale were modified because of cultural, semantic or contextual inappropriateness. The ABC-S scale demonstrated very good intra- and inter-rater reproducibility, with an average intraclass correlation coefficient (ICC) of 0.85 and 0.81, respectively. The self-perceived balance confidence among elderly Sepedi individuals, as evaluated by the ABC-S scale, was high, with an average score of 81.3 and a range of 58.1 to 95.9.

**Conclusion:**

The ABC-S scale is a reliable measurement tool to investigate balance confidence in Sepedi-speaking older adults.

**Contribution:**

The ABC-S scale is a valuable screening tool for the identification of balance difficulties in Sepedi-speaking older adults as well as research settings.

## Introduction

Population ageing is a global phenomenon marked by an unprecedented growth of the elderly population (World Health Organization [WHO], 2022). The United Nations (2015a) observes that this population is growing more rapidly in developing countries compared to developed nations. Supporting this, the WHO (2022) predicts that four-fifths of older adults will be living in low- and middle-income countries in 2050, totalling approximately 1.2 billion elderly individuals (United Nations, 2019). In South Africa, the percentage of elderly people is on the rise, with the growth rate among those aged 60 years and older increasing from 1.1% in the year 2002–2003 to 3.0% in the year 2019–2020 (Statistics South Africa [Stats SA], 2020a). The rise is attributed to decreased fertility and increased longevity (Solanki et al., 2019), posing significant challenges to health and social systems, which are unprepared to address the chronic medical conditions associated with population ageing (WHO, 2022).

To enhance the quality of life of older adults and ensure that longer life is associated with positive experiences, the WHO (2002) suggests prioritising opportunities related to health, participation and security, a process known as ‘active ageing’ (p. 12). However, falls^1^ and their related injuries often lead to fear of falling, loss of independence, institutionalisation (Khow & Visvanathan, 2017) and disability (Appeadu & Bordoni, 2023) and thus pose a threat to active ageing. Evidence suggests that near falls^2^ are significant predictors of subsequent falls (Nagai et al., 2017), with falls accounting for the second most injury-related mortalities among the elderly globally (WHO, 2021).

An estimated 684 000 older adults worldwide suffer fatal falls annually (WHO, 2021). The global prevalence of falls among older adults is estimated to be 26.5% based on a systematic review of 104 articles (Salari et al., 2022). These statistics align with a study conducted in Cape Town, South Africa, where the prevalence rate of falls among the elderly was estimated to be 26.4% (Kalula et al., 2016). The United Nations (2015b) projects that the population of older adults in South Africa will double from 7.7 million in 2015 to 15.4 million in 2050, making falls a matter of concern.

Falls are complex events that result from the interactions between several risk factors (Chiarella et al., 2020). Elderly individuals face an increased risk of falling because of physical, sensory and cognitive changes associated with ageing (Montero-Odasso et al., 2022; WHO, 2021). Khow and Visvanathan (2017) categorise risk factors into two main groups: intrinsic and extrinsic factors. Intrinsic risk factors are specific to each individual and include functional abilities and comorbidities, such as the co-occurrence of hearing loss and vestibulopathy (Chiarella et al., 2020). Extrinsic risk factors, on the other hand, are external hazards such as dilapidated infrastructure contributing to the high prevalence rate of falls in South Africa (Kalula et al., 2016).

Despite national guidelines on fall prevention, there are no published data indicating their implementation in clinical practice (Kalu et al., 2019). In addition, there is a lack of preventative initiatives and educational programmes offered to the public in South Africa (Kalula et al., 2016), a concern given the potential role of various healthcare professionals in identifying individuals at risk of falling or with a fear of falling (De Clercq et al., 2020).

Audiologists, trained in the assessment and rehabilitation of vestibular problems, are well placed to assess fall risk, particularly because evidence suggests a link between hearing loss in older adults and increased fall risk (Jiam et al., 2016; Rogers, 2021). Audiologists, because of their access to older adults with hearing loss and vestibular problems, should thus play an integral role in screening of fall risk (Rogers, 2021), a critical step towards prevention of falls (Khow & Visvanathan, 2017). Despite this, the role of audiologists in fall risk assessment is not consistently emphasised across various countries’ audiology scope of practice documents (Van Rie et al., 2022). There are a wide variety of screening measures to identify possible fall risk. A recent review conducted by Van Rie and colleagues (2022) reported that the four most reported fall risk screening measures used by audiologists, in order of frequency, were case history, the Timed Up and Go (TUG) test (Podsiadlo & Richardson, 1991), the Activities-specific Balance Confidence (ABC) scale (Powell & Myers, 1995) and the Dizziness Handicap Inventory (DHI) (Jacobson & Newman, 1990).

**FIGURE 1:**
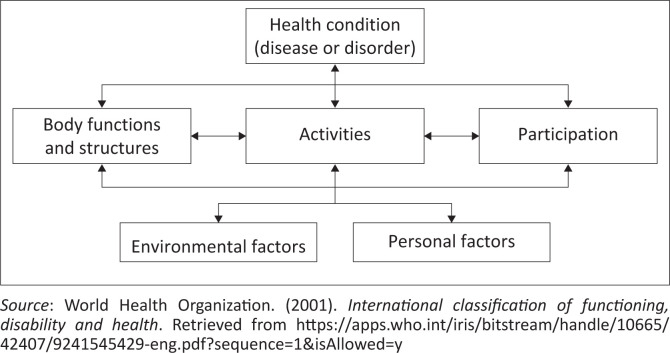
International Classification of Functioning, Disability and Health.

The 16-item ABC scale, developed by Powell and Myers (1995), is a reliable and valid (Botner et al., 2005; Huang & Wang, 2005; Salbach et al., 2006) measure widely used across multiple professions to identify older adults who may be at risk of falling by quantifying their self-perceived level of balance confidence in executing activities of daily living without falling or becoming unsteady (Sakakibara et al., 2011; Van Rie et al., 2022). In addition to use with community-dwelling elderly, the ABC scale is suitable for use with, among others, individuals with stroke (Botner et al., 2005; Seamon et al., 2019), vestibular disorders (Oh et al., 2023), persons with dystonia (Boyce et al., 2017) and Parkinson’s disease (Franchignoni et al., 2014). This scale is quick to administer and correlates highly with the TUG, a functional mobility test, demonstrating the relationship between balance confidence and functional mobility (Hatch et al., 2003). The ABC scale further measures constructs within the International Classification of Functioning, Disability and Health (ICF) framework (Figure 1). The ICF is an internationally recognised framework that is underpinned by the biopsychosocial model of disability (Dantas et al., 2020). This comprehensive framework provides a scientific basis and common language for healthcare professionals worldwide to describe and classify falls, their associated risk factors in older adults (De Clercq et al., 2021), causes of falls and interventions aimed at fall prevention (Baris & Seren Intepeler, 2019).

In the activity domain, balance and falls gait (including stairs) and transfers are measured, while in the participation domain, it focuses on community function, home management, leisure and recreational activities role function and shopping (Academy of Neurologic Physical Therapy, 2013). This is important, as a recent study has found that fall assessment should focus on the activities and participation domains as this may decrease the risk of falling in older adults (De Clercq et al., 2021). These findings are supported by Johnson (2018) who explains that ‘increased knowledge of the activities that are linked to falls and common fall-related injuries could be a valuable contribution to the prevention of falls in community-dwelling older adults’ (p. 22).

The ABC scale has been adapted in other countries, such as Brazil (Marques et al., 2013), China (Hsu & Miller, 2006), Germany (Schott, 2008) and India (Moiz et al., 2017), among others. While the utility of the ABC scale has been recognised globally, it does not account for variations across different contexts, cultures or languages. South African citizens have the right to access healthcare services in a language of their choice (Van den Berg, 2016). In the South African context, only one such adaptation (Kamanji, 2016) has occurred. Currently, no measures are available for assessing balance confidence in older Sepedi-speaking adults. When such measures are not available, health professionals may feel compelled to self-translate balance confidence scales, which may affect the reliability thereof (Sahu & Kulkarni, 2017).

In South Africa, Sepedi is the third largest African language, the fifth most spoken language in the country and the first language of approximately 4.6 million people (Stats SA, 2011). The majority (52.9%) of first language Sepedi speakers live in the Limpopo province. Sepedi is also spoken by 10.6% of people who live in Gauteng and 9.3% of people who live in Mpumalanga (Stats SA, 2011). Therefore, the aim of this study was to translate and culturally adapt the ABC scale into Sepedi, as well as determine the reliability of the Sepedi version of the ABC (ABC-S) scale. The secondary aim was to determine the self-perceived balance confidence of first language Sepedi speakers in a rural community in Limpopo.

## Methodology

### Research design

A mixed-methods research approach was employed for this two-phased study. In the first phase, a formative qualitative methodology was used to translate and culturally adapt the ABC scale (WHO, 2016). In the second phase, a descriptive research design was used to determine the reliability of the ABC-S scale and describe the self-perceived balance confidence among first language Sepedi speakers in a rural community in Limpopo.

### Context

The second phase of this study was conducted in a rural area of the Greater Sekhukhune District Municipality of the Limpopo province, where Sepedi is the predominant language spoken, accounting for 83% of the population (Integrated Development Plan, 2021).

### Participants

Using purposive sampling, a total of 32 participants were recruited for the second phase of the study. Participants were required to meet the following criteria: (1) be between the ages of 60 and 88 years (Kamanji, 2016; Marques et al., 2013) to allow for comparison with the isiZulu (Kamanji, 2016) and Brazilian (Marques et al., 2013) versions of the scale; (2) be first language Sepedi speakers; (3) reside in a rural community; (4) be ambulatory and capable of independently performing daily activities inside and outside of the home either with or without walking aids; and (5) be able to understand and follow multistep instructions. Individuals with medical conditions that increase the risk of falling (e.g. neurological disorders, musculoskeletal disorders, orthostatic hypotension, etc.) as well as those with unmanaged hearing and visual impairments were excluded from the study.

The mean age of participants was 70.06 years (±7.26; range: 60–88) and the majority were female (*n* = 84%). The gender distribution aligns with the distribution of the broader Sekhukhune District Municipality population where 65% of the population in this age group is female (Integrated Development Plan, 2021).

### Measures

Data collection involved the use of a self-developed case history questionnaire (Appendix 1) and the ABC-S scale. The case history questionnaire consisted of 19 questions organised in four sections: demographic information, balance history, medical history and family history. The development of the case history questionnaire was guided by literature (Kamanji, 2016). The ABC-S scale has 16 items rated on an 11-point subscale ranging from 0% (no confidence) to 100% (completely confident) with increments of 10%. Scores below 50% indicate a low level of physical functioning. Scores above 80% represent a high level of physical functioning (Marques et al., 2013). Scores of <67% have shown to be predictive of future falls (Reelick et al., 2009).

### Data collection

#### First phase

The first phase of data collection involved the cultural adaptation and translation of the ABC scale into Sepedi, following a five-step process as outlined by the WHO (2016). This process included forward translation, expert panel assessment, back translation, pretesting and final version development.

#### Forward translation

The ABC scale was translated into Sepedi by a translation agency with 26 years’ experience in healthcare and medical translations. All translators hired by the agency are native speakers with the relevant credentials. The translator, appointed by the agency, was a first language Sepedi speaker proficient in English.

#### Expert panel assessment

The expert panel comprised three health professionals (two nurses and one pharmacist) with an undergraduate degree and a minimum of 3 years’ experience in working with older adults in rural communities. They were purposively selected from a health facility in the area and resided in the community where the study was conducted. They were requested to rate the cultural and contextual relevance of each activity assessed in the scale to the rural South African context. Each question was rated for relevance, with scores ranging from 1 (highly irrelevant) to 5 (highly relevant). For questions judged to be culturally or contextually irrelevant (ratings of 1 and 2), panel members were required to provide an explanation and a more culturally or contextually relevant alternative. Items with a score of 4 (relevant) and 5 (highly relevant) were considered culturally and contextually relevant. An inter-judge percentage agreement of ≥80% was used (McHugh, 2012).

#### Back translation

The culturally adapted ABC-S scale was translated back into English by an independent translator (proficient in Sepedi and English) from the same translation agency used in the forward translation.

#### Pretesting

The ABC-S scale was piloted on three female participants, aged 65, 72 and 80 years, respectively, and who met the same inclusion criteria as for the main study. The objectives of the pilot study were to determine the semantic, contextual and cultural appropriateness of each question in the ABC-S scale. Eight modifications were made based on the feedback from the pilot study participants that led to the final version of the ABC-S scale (Appendix 2).

#### Second phase

During the second phase, two research assistants were trained to recruit participants, complete the case history questionnaire and administer the ABC-S scale. The research assistants were employed as hearing screeners in the community where the study was conducted and had at least 4 years’ experience in case history taking. They were both first language Sepedi speakers proficient in English. Potential participants were recruited at two of the local primary healthcare clinics using purposive sampling techniques. Only those who provided written informed consent were included in the study. Data collection occurred over an 8-week period, either at participants’ homes or community sites based on their preferences. The ABC-S scale was administered by the two research assistants during the initial data collection period and readministered 2 weeks later by rater one to assess inter-rater and test-retest reliability.

Data were encoded and analysed using appropriate statistical techniques. The Cronbach’s alpha coefficient and item-total correlations were utilised to assess the internal consistency of the ABC-S scale. Test-retest reliability and inter-rater reliability were determined using the intraclass correlation coefficient (ICC). Descriptive statistics, including mean, range and standard deviation, were used to describe the self-perceived balance confidence.

### Ethical considerations

Ethical clearance to conduct this study was obtained before data collection from the University of the Witwatersrand School of Human and Community Development Ethics Committee (No. STA_2021_5). The research adhered to the ethical principles outlined in the World Medical Association Declaration of Helsinki (World Medical Association [WMA], 2013). Permission to conduct the study was obtained from relevant authorities. Participants were fully informed of the nature of the study as well as their rights and assured of anonymity and confidentiality. Participants identified as having a fall risk were referred to their closest audiology department for assessment. Three options were available: the local community-based audiology clinic or two hospitals in the Greater Sekhukhune District Municipality. The two hospitals also offer physiotherapy services.

## Results and discussion

The aims of this study were to translate and culturally adapt the ABC scale into Sepedi as well as determine the reliability of the ABC-S scale. In addition, the self-perceived balance confidence of first language Sepedi speakers in a rural community in Limpopo was determined.

### Cross-cultural adaptation of the translated Activities-specific Balance Confidence scale

The translated ABC scale (Appendix 2) was culturally adapted by a bilingual expert panel to suit the needs of the rural South African context. Ten of the original 16 items were modified to ensure semantic, contextual and cultural appropriateness. These modifications are documented in Table 1.

**TABLE 1:** Original and modified items in the cross-cultural adaptation of the Activities-specific Balance Confidence scale.

Item no.	Original items of the ABC scale	Modified items of the ABC-S scale
3	Bend over and pick up a slipper from the front of a closet floor	Bend over and pick up a *shoe* from the *area where one stores their belongings*
4	Reach for a small can off a shelf at eye level	Reach for a small *tin that is on top of a shelf that you can see*
8	Walk outside the house to a car parked in the driveway	Walk outside to a *vehicle* in the *yard*
9	Get into or out of a car	Get into or out of a *vehicle*
10	Walk across a parking lot to the mall	Walk across a parking lot to the mall or shops
11	Walk up or down a ramp	Walk up or down a *hill*
12	Walk in a crowded mall where people rapidly walk past you	Walk in a crowded mall or shop where people rapidly walk past you
13	Are bumped into by people as you walk through the mall	Are bumped into by people as you walk through the mall or shops
15	Step onto or off an escalator while holding onto parcels such that you cannot hold onto the railing	Step onto or off an escalator while holding onto *luggage* such that you cannot hold onto the railing
16	Walk outside on icy sidewalks	Walk outside on *surfaces largely covered by dew*

ABC, Activities-specific Balance Confidence; ABC-S, Sepedi version of the ABC.

The adjustments were necessary because of the distinct local reality and cultural differences of the South African participants, considering that the original scales development is based on the Canadian population (Powell & Myers, 1995).

For instance, item 3 originally ‘pick up a slipper from the front of the closet floor’ underwent modifications to ‘pick up a shoe from the area where one stores their belongings’ to better align with local terminology and cultural context. Specifically, the terms ‘slipper’ and ‘closet’ were not familiar to the South African participants. Marques et al. (2013) caution that using unfamiliar terminology may hinder comprehension. None of the participants reported any doubts as to the meaning during the administration of the ABC-S scale. The adaptations aimed to ensure comprehension without altering the activity’s intended meaning, as evidenced by comparable scores to the Brazilian-Portuguese and Canadian studies (Table 5).

Similarly, item 4 was also adapted by changing the phrase ‘reach for a small can off a shelf at eye level’ to ‘reach for a small tin that is on top of a shelf that you can see’ to align with the common expression in the Sepedi culture.

Items 8 and 9 were adapted to account for socioeconomic differences, replacing ‘walk outside the house to a car parked in the driveway’ with ‘walk outside to a vehicle in the yard’ emphasising ‘vehicle’ to accommodate varied transportation modes. Most of the residents in Limpopo use taxis (Stats SA, 2020b), as cars are owned by less than 15% of households in rural communities (Stats SA, 2018). Driveways are also uncommon in rural communities.

To align with the contextual realities of the Elias Motsoaledi Local Municipality (EMLM) area and the lifestyle of its residents, the term ‘shop(s)’ was added to items 10, 12 and 13. Most of the people who reside in the EMLM area and other rural areas in South Africa go to shops, which are more accessible to them compared to malls.

During the pilot study, participants expressed unfamiliarity with the term ‘ramp’ in item 11 (‘walk up or down a ramp’), leading to its modification ‘walk up or down a hill’. In item 15, the word ‘parcels’ was replaced with ‘luggage’ to better suit the context of the study population.

Item 16 (‘walk outside on icy sidewalks’) underwent the final modification to account for differences in climate, as snow is extremely rare in South Africa. The term ‘sidewalk’ was also changed. Item 16 was thus changed to ‘walk outside on a surface largely covered by dew’ to reflect context and align with the Sepedi expression.

These meticulous adaptations underscore the significance of tailoring assessment tools for cultural relevance, ensuring the reliability of the ABC-S scale in the local South African setting.

### Reliability of the Sepedi version of the ABC scale

The test-retest reliability of the ABC-S scale is shown in Table 2. The ICC for the total score exhibited a high level of reliability, measuring at 0.85, with a 95% confidence interval. This is consistent with the results obtained for the isiZulu ABC scale, which demonstrated a score of 0.82 (Kamanji, 2016) and balance confidence in individuals with stroke at 0.85 (Botner et al., 2005). Other versions of the ABC scale have demonstrated excellent test-retest reliability, such as the Brazilian-Portuguese (Marques et al., 2013), Hindi (Moiz et al., 2017) and Chinese (Hsu & Miller, 2006) versions with scores of 0.94, 0.97 and 0.99, respectively.

**TABLE 2:** Test-retest reproducibility: Mean (standard error) and intraclass correlation coefficient of the Sepedi version of the Activities-specific Balance Confidence scale.

ABC-S scale items	Evaluation 1		Evaluation 2	
	Mean†	SE	Mean‡	SE
1	80	20.0	97.5	2.5
2	67.5	6.3	52.5	22.1
3	65	15.6	60	19.6
4	70	10.0	72.5	24.3
5	57.5	13.8	62.5	22.5
6	30	17.8	20	9.1
7	85	2.9	90	4.1
8	92.5	4.8	97.5	2.5
9	87.5	7.5	100	0.0
10	75	16.6	77.5	16.5
11	75	10.4	57.5	19.3
12	55	11.9	80	16.8
13	67.5	16.5	80	16.8
14	55	20.2	22.5	22.5
15	45	18.5	20	20.0
16	100	0.0	95	5.0

**Total**	**1107.5**	-	**1085.0**	-

Note: Average ICC = 0.85.

ICC, intraclass correlation coefficient; ABC-S, Sepedi version of the Activities-specific Balance Confidence scale.

† average = 69.2;

‡ average = 67.8.

Table 3 shows the inter-rater reliability of the ABC-S scale. The ICC for the total score exhibits a high level of reliability, measuring at 0.81 with a confidence interval of 95%. This is consistent with other versions of the ABC scale such as the Brazilian-Portuguese translation, with a score of 0.80 (Marques et al., 2013). These findings suggest that the scale produces consistent results over time and between different raters and can be used in clinical and research settings.

**TABLE 3:** Inter-rater reproducibility: Mean (standard error) and intraclass correlation coefficient of the Sepedi version of the Activities-specific Balance Confidence scale.

ABC-S scale items	Rater 1		Rater 2	
	Mean†	SE	Mean‡	SE
1	83.75	8.9	56.25	11.3
2	87.50	7.7	77.50	8.0
3	88.75	5.2	82.50	5.9
4	85.00	8.9	75.00	7.6
5	87.50	6.8	80.00	6.6
6	76.25	7.3	67.50	6.5
7	96.25	2.6	87.50	4.9
8	95.00	3.8	87.50	5.6
9	100.00	0.0	95.00	1.9
10	96.25	2.6	90.00	4.2
11	95.00	2.7	88.75	5.5
12	97.50	2.5	88.75	4.8
13	97.50	1.6	87.50	3.1
14	85.00	5.0	75.00	6.8
15	82.50	7.3	71.25	7.7
16	100.00	0.0	92.50	3.1

**Total**	**1453.75**	-	**1313.75**	-

Note: Average ICC = 0.81.

ICC, intraclass correlation coefficient; ABC-S, Sepedi version of the Activities-specific Balance Confidence scale.

† average = 90.86;

‡ average = 81.41.

Table 4 shows the internal consistency of the ABC-S scale, which was determined using the Cronbach’s alpha coefficient and item-total correlations. The scale showed a high internal consistency of α = 0.92. This was reinforced by moderate-to-strong item-total correlations for 13 of the items, ranging from 0.41 to 0.85 and exceeding the cutoff value of 0.70 (Ferketich, 1991).

**TABLE 4:** Internal consistency of the Sepedi version of the Activities-specific Balance Confidence scale.

Item no.	Corrected item-total correlation	Alpha, if item deleted
1	0.26	0.92
2	0.68	0.91
3	0.29	0.92
4	0.66	0.91
5	0.74	0.91
6	0.76	0.91
7	0.65	0.91
8	0.64	0.91
9	0.65	0.91
10	0.41	0.92
11	0.85	0.90
12	0.68	0.91
13	0.62	0.91
14	0.77	0.90
15	0.78	0.90
16	0.30	0.92

An α ≥ 0.9 is considered excellent (Bland & Altman, 1997). This indicates that the items of the ABC-S scale measure a single, unidimensional construct (Tavakol & Dennick, 2011) and is thus appropriate for use in clinical and research settings. The internal consistency of the ABC-S scale is consistent with findings reported in previous studies (see Guan et al., 2012; Kamanji, 2016; Mak et al., 2007; Powell & Myers, 1995; Salbach et al., 2006; Schott, 2008; Van Heuvelen et al., 2005). Subsequent analysis indicated that the majority of the items are suitable for retention, leading to a decrease in the alpha coefficient if they are excluded. Nevertheless, there were four exceptions to this trend, specifically items 1, 3, 10 and 16, for which the removal would result in an increased alpha coefficient of 0.92. Therefore, it is advisable to consider removing these items. This aligns with the observed item-total correlations for items 1, 3 and 16, which demonstrated a weak correlation. According to Powell and Myers (1995), the internal consistency of the scale does not exhibit a significant improvement when a few items are removed. Thus, the deletion of item 16, which pertains to ‘walking outside on icy sidewalks’, should not pose any challenges for administering the scale in warmer climates, such as South Africa (Myers et al., 1998).

### Self-perceived balance confidence of elderly people

The mean confidence level of first language Sepedi speakers in a rural community in the Limpopo province was 81.3 (±10.3; range: 58.1–95.9) (Table 5). This is suggestive of a high level of self-perceived confidence in their ability to maintain balance during various daily activities (Myers et al., 1998).

**TABLE 5:** Comparison of the Sepedi version of the Activities-specific Balance Confidence score with the Brazilian, isiZulu and the original English version.

Item no.	ABC-S score		Brazilian ABC score		isiZulu ABC score		Original ABC score	
	(*n* = 32)		(*n* = 40)		(*n* = 32)		(*n* = 60)	
	Mean	SD	Mean	SD	Mean	SD	Mean	SD
1	89.4	-	88.2	-	74.2	-	87.5	-
2	80.0	-	85.5	-	48.8	-	64.8	-
3	87.5	-	86.2	-	57.9	-	62.8	-
4	77.8	-	93.5	-	60.6	-	89.5	-
5	79.7	-	80.5	-	47.9	-	46.5	-
6	58.1	-	68.5	-	26.9	-	38.0	-
7	88.4	-	96.7	-	63.0	-	66.8	-
8	89.7	-	87.2	-	72.7	-	71.6	-
9	90.6	-	86.5	-	68.2	-	78.5	-
10	83.4	-	89.7	-	48.5	-	67.7	-
11	81.6	-	81.5	-	41.2	-	61.0	-
12	82.2	-	84.0	-	51.2	-	62.2	-
13	84.1	-	79.5	-	41.2	-	53.1	-
14	67.2	-	76.7	-	49.1	-	52.3	-
15	64.4	-	63.2	-	28.8	-	31.3	-
16	95.9	-	61.2	-	36.1	-	20.7	-

**Total score**	**81.3**	**10.3**	**81.7**	**10.1**	**51.0**	**14.4**	**59.6**	**27.7**

Source: Adapted from Kamanji, R. (2016). Zulu translation and cross-cultural adaptation of the activities-specific balance confidence (ABC) scale. Research report. University of the Witwatersrand; Marques, A.P., Mendes, Y.C., Taddei, U., Pereira, C.A., & Assumpção, A. (2013). Brazilian-Portuguese translation and cross-cultural adaptation of the activities-specific balance confidence (ABC) scale. Brazilian Journal of Physical Therapy, 17(2), 170–178. https://doi.org/10.1590/S1413-35552012005000072; Powell, L.E., & Myers, A.M. (1995). The activities-specific balance confidence (ABC) scale. The Journals of Gerontology Series A: Biological Sciences and Medical Sciences, 50A(1), M28–M34. https://doi.org/10.1093/gerona/50A.1.M28

Note: Items were rated from 0% (no confidence) to 100% (complete confidence).

ABC-S, Sepedi version of the ABC; ABC, Activities-specific Balance Confidence.

Subjective fear of falling was assessed through the unvalidated single-item question: ‘Do you have a fear of falling?’ asked during case history. Of the 12 participants who indicated fear of falling, 6 (50%) obtained a total score of <67, with scores ranging from 23.13 to 65. Only two participants (6.25%) who did not report a fear of falling obtained scores below 67. The single-item question was significantly correlated to the ABC scores in the sample (r = −0.567).

The two items with the lowest degrees of confidence in this study were identical to the findings in the Brazilian (Marques et al., 2013), isiZulu (Kamanji, 2016) and Canadian populations (Powell & Myers, 1995). These items generally involve activities that pose greater challenges or perceived risks for the participants. These include item 6 (‘stand on a chair and reach for something’) and item 15 (‘step onto or off an escalator while holding onto parcels such that you cannot hold onto the railing’). These items had values ranging from 58.1 to 64.4, consistent with findings from other studies (Botner et al., 2005; Mak et al., 2007; Salbach et al., 2006; Schepens et al., 2010). Item 16 in this study, however, had the highest score (95.9), in contrast to the Canadian and Brazilian population at 20.7 and 61.2, respectively. This difference may be explained by the perceived level of difficulty walking on a surface covered by dew (this study) in comparison to walking on ice.

A score lower than 67% has been identified as an indicator of fall risk (Lockhart et al., 2019; Reelick et al., 2009). When comparing the mean scores across different cultural and linguistic contexts, it is evident that the Sepedi (81.3) and Brazilian-Portuguese (81.7) studies yielded considerably higher mean scores compared to the isiZulu (51) and Canadian (59.6) studies, as shown in Table 5. In both the Sepedi and Brazilian-Portuguese studies, only three items obtained scores lower than 67%, indicating a relatively low fall risk among the participants. This finding is in stark contrast to the isiZulu (Kamanji, 2016) and Canadian (Powell & Myers, 1995) versions of the ABC scale, where 13 and 12 items, respectively, received scores below the fall risk threshold. These results suggest that elderly individuals in the Sepedi and Brazilian-Portuguese-speaking populations demonstrated a higher level of self-perceived balance confidence when compared to their Canadian counterparts, and a lower likelihood of falling when compared to their isiZulu and Canadian counterparts.

The disparity on fall risk among these populations may be attributed to differences in the selection criteria employed in the original and isiZulu studies. In a study conducted by Lajoie and Gallagher (2004), individuals who had experienced a stroke or had preexisting decreased postural control were included as participants. This selection criterion likely contributed to lower balance confidence scores in the Canadian study population. Similarly, the isiZulu study by Kamanji (2016) included participants with physical impairments. These impairments may have negatively impacted balance confidence levels, resulting in the comparatively lower mean scores observed in the isiZulu study. In contrast, the current and Brazilian-Portuguese studies (Marques et al., 2013) excluded individuals with medical conditions that increase the risk of falling and those with unmanaged hearing and visual impairments. Consequently, the participants in these studies may have had a higher baseline level of balance confidence, leading to the elevated mean scores observed.

## Conclusion

The adapted ABC-S scale demonstrated high internal consistency, test-retest reliability and inter-rater reliability. The findings of this study support the recommendation for the use of the ABC-S scale in Sepedi-speaking older adults to assess their self-perceived level of balance confidence. It is a valuable tool for clinicians, researchers and policymakers working in the field of fall prevention that can contribute to the development and implementation of effective preventative initiatives, ultimately improving the overall well-being and quality of life of Sepedi-speaking older adults. Furthermore, the cultural adaptation of the ABC scale into Sepedi enhances its applicability and ensures that it resonates with the linguistic and cultural contexts of the target population. This consideration is crucial in promoting inclusivity and sensitivity to diverse cultural backgrounds (Pascoe & Norman, 2011) ultimately leading to more accurate and meaningful assessments of balance confidence.

A limitation of the study is the small sample size and the recruitment of participants from only one municipal area in the Limpopo province, which limits the generalisability of the study. Further studies should explore the validity of the tool as well as the replication of the study across the different provinces and contexts (e.g., urban vs. rural) where significant numbers of Sepedi-speaking individuals reside as well as medical conditions. This will assist with the generalisation of the results. The ABC scale should also be translated and culturally adapted into other indigenous South African languages.

## Footnotes

1 A fall is defined as an event that results in a person inadvertently coming to rest on the ground or floor or other lower level (WHO, 2021).

2 A near fall, as defined by Maiden and colleagues (2014), is ‘a stumble event or loss of balance that would result in a fall if sufficient recovery mechanisms were not activated’ (p. 645).

## Appendix 1: Case history questionnaire

### Participant particulars

Participant number: ________ Gender: ________ Age: __________

Home language: ___________________Date: ________________

**Please complete the following** (please check [√] the box that applies to you)

Marital status

□ Single    □ Married    □ Divorced    □ Widowed

Highest level of education

□ No formal education    □ Grade 1–7    □ Grade 8–9

□ Grade 10–12    □ College    □ University

### Balance history

1. Do you have a fear of falling?
  □ Yes
  □ No
2. Do you use walking aids indoors? If yes, please describe.
  □ Yes_____________________________________________________
  □ No
3. Do you use walking aids outdoors? If yes, please describe.
  □ Yes_____________________________________________________
  □ No
4. Do you need physical assistance when walking outside? If yes, please explain what assistance you require, for example: holding onto someone’s arm.
  □ Yes_____________________________________________________
  □ No
5. Have you felt unsteady on your feet in the last 6 months?
  □ Yes
  □ No
6. Have you fallen anytime in the last 6 months? If yes, please indicate how many times you have fallen.
  □ Yes_____________________________________________________
  □ No
7. How many times do you exercise per week?
  □ Less than three times
  □ Three times or more
8. Please list what exercise you do below and for how long.
  □ ___________________________________________________________
  □ ___________________________________________________________
  □ ___________________________________________________________

### Medical history

9. Please list any allergies that you have.
  □ ___________________________________________________________
  □ ___________________________________________________________
  □ ___________________________________________________________
10. Please list any medical conditions that you have.
  □ ___________________________________________________________
  □ ___________________________________________________________
  □ ___________________________________________________________
11. Please list the medication that you are currently taking.
  □ ___________________________________________________________
  □ ___________________________________________________________
  □ ___________________________________________________________
12. Please list any surgeries or operations that you have had.
  □ ___________________________________________________________
  □ ___________________________________________________________
  □ ___________________________________________________________
13. Have you had any previous medical examination or testing done? (Hearing, vision, x-rays, head scans etc.). If yes, please provide details as well as a summary of the results if possible.
  □ Yes _______________________________________________________
  □ No

### Family history

14. Please indicate if you have a family history of any of the following by ticking the box.
  □ Hearing loss
  □ Dizziness
  □ High blood pressure
  □ Low blood pressure
  □ Headaches
  □ Migraines
  □ Diabetes
  □ Panic attacks

## Appendix 2: Sepedi version of the ABC (ABC-S) scale

Kelo ya mešongwana- yeo e ikgethilego ya boitshepo bja palantshe (ABC)

Go mošongwana o mongwe le o mongwe woo o latelago, kgopela o laetše maemo a gago a boitshepo ka go kgetha nomoro ya maleba go ya le ka maemo a kelo:

0% 10 20 30 40 50 60 70 80 90 100%

*go hloka boithsepo      go ba le boitshepo*

‘Ona le boitshepo bjo bo kakang ba gore o ka se lahlegele ke palantshe goba go tšhekinyega ge o…

1. …ge o sepela ka gae? ____%
2. …o eya godimo le fase disetepiseng? ____%
3. …ge o khunama o topa seetabolaong fase ge o tloga ka pele ga bo bolokelo bja phaahlo? ____%
4. …ge o obeletša kotikoti ye nnyane godimo ga šelefe yeo o kgonago go e bona? ____%
5. …go tsatsampele gore o kgone go fihlelela seo se lego ka godimo ga hlogo ya gago? ____%
6. …go namela setulo go ka kgona go fihlelela se sengwe? ____%
7. …go swiela? ____%
8. …go sepela ka ntle o eya ka jareteng mo go emago sefatanaga? ____%
9. …go namela le go fologa sefatanaga? ____%
10. …o tloga bo dulong bja difatanaga go ya mmolong/mabenkeleng? ____%
11. …go namela le go fologa mototolo? ____%
12. …o sepela ka mmolong/mabenkeleng moo batho e lego ba bantši ba fetafeta kgauswi le wena? ____%
13. …go hlakahlakana le batho ge o sepela ka mmolong/mabenkeleng?____%
14. … o namela setepisitshepetšo o itshwareleditše? ____%
15. … o namela goba o fologa setepisitshepetšo o swere merwalo yeo e ka go paledišago go itshwareletša? ____%
16. …ge o sepela ka ntle go dimo ga phoka?____%

### Instructions for scoring

The ABC is an 11-point scale and ratings should consist of whole numbers (0–100) for each item. Total the ratings (possible range = 0–1600) and divide by 16 to get each person’s ABC score.

Total score: _______________________
